# Supplementary material for: MetaRibo-Seq measures translation in microbiomes
Source: Nat Commun. 2020 Jun 29;11:3268. doi: 10.1038/s41467-020-17081-z (PMC7324362; doi:10.1038/s41467-020-17081-z)
Supplement: Supplementary file 10 — Supplementary Data 7 [file 41467_2020_17081_MOESM10_ESM.zip › File2/Confidence_High_Taxonomy/296937_out.krona.html]

Javascript must be enabled to view this page.

members
magnitude
magnitudeUnassigned
count
unassigned
taxon
rank

296937\_out

3

2
3
superkingdom

1239
3
phylum

class
3
186801

186802
3
order

family
2
31979

2
genus
1649459


SRS022713\_contig\_number\_5255SRS057478\_contig\_number\_contig-100\_3897.47914
species
2
154046

family
1
186803

genus
1
1506553

1
84030
species

SRS023829\_contig\_number\_contig-100\_1403.1403
